# Effect of the supplementation of virgin coriander seed oil on reducing reactivity in healthy women with sensitive skin: a randomized double-blind placebo-controlled pilot clinical study

**DOI:** 10.29219/fnr.v66.7730

**Published:** 2022-03-23

**Authors:** Catherine Kern, Christian Gombert, Alicia Roso, Christine Garcia

**Affiliations:** Seppic Research and Innovation, La Garenne Colombes, France

**Keywords:** sensitive skin, coriander seed oil, soothing, stripping, stinging, pilot clinical study

## Abstract

Sensitive skin is a common condition that affects many people in the world, especially women. This syndrome is defined by the occurrence of unpleasant sensations such as stinging and burning in response to stimuli that should not normally provoke such sensations. Coriander seed oil (CSO) is a 100% virgin oil of coriander seeds and boasts a specific composition of fatty acids, mainly petroselinic acid (60–75%). It has demonstrated its ability to regulate inflammation (NF-κB pathway) and nociception (TRPA1 pathway), two mechanisms supporting sensitive skin, in previous *in vitro* research. It was, therefore, a good candidate to be tested *in vivo* on sensitive skin conditions. A pilot clinical study was conducted to evaluate the effect of this ingredient on healthy women showing excessive skin reactions, mainly redness and discomfort when subjected to external stress. The results showed that the daily consumption of 200 mg of CSO for 28 days effectively reduced redness induced by stripping stress and itching induced by stinging stress. It also improved the perception of skin sensitivity and reactivity after 56 days of consumption. These clinical results confirmed that CSO is a promising ingredient to contribute to reducing reactivity in sensitive skin.

## Popular scientific summary

- Sensitive skin is a common condition that affects many people in the world.
- Coriander seed oil is a 100% virgin oil of coriander seeds and has already shown positive effects *in vitro* on mechanisms of action associated with sensitive skin.
- Daily consumption of 200 mg of coriander seed oil for 28 days effectively reduces reactivity in women with sensitive skin.

Sensitive or reactive skin is a common condition that affects approximately 50% of people: 60% of women and 40% of men. The prevalence of sensitive skin varies among countries and has increased over the past decades (1, 2). Recently, a consensus was reached by a working group (special interest group on sensitive skin of the International Forum for the Study of Itch) to use the following definition: ‘A syndrome defined by the occurrence of unpleasant sensations (stinging, burning, pain, pruritus, and tingling sensations) in response to stimuli that should not normally provoke such sensations. These unpleasant sensations cannot be explained by lesions attributable to any skin disease. The skin can appear normal or be accompanied by erythema. Sensitive skin can affect all body locations, especially the face’ (2). While the face is the most common site of sensitive skin, other parts of the body are also affected, such as hands, scalp, feet, neck, torso, and back (3). The sensations described can vary widely: pain, pruritus, burning, tingling, prickling, pungency, thickening, or dryness of the skin, and these may or may not be accompanied by signs such as redness, mild erythema, telangiectasias, xerosis, desquamation, or urticaria. Environmental factors, such as UV irradiation, air pollution, and climate conditions (temperature, humidity, wind, sun exposure, etc.); lifestyle factors, such as cosmetic usage, diet, and alcohol consumption; and physiological factors, such as stress or endogenous hormones, have been reported to induce or worsen the symptoms of sensitive skin (4, 5). The main mechanisms supporting sensitive skin are the dysfunction of the epidermal barrier leading to increased inflammation and a neurosensory dysfunction leading to symptoms such as burning, tingling, stinging, pain, and itching.

Cutaneous fatty acids found in the epidermis are key determinants of skin health as they can be metabolized into various mediators that regulate epidermis homeostasis, including barrier function and inflammation regulation (6). They can be influenced by diet, especially the consumption of fatty acids and oils. Coriander is an annual herb from the Apiaceae (Umbelliferae) family that originates from the Near East and Mediterranean area. It is a well-known medicinal herb and has been shown to exhibit antioxidant (7), antimicrobial (8), and anti-inflammatory activities (9). Coriander seed oil (CSO) is a 100% virgin oil of *Coriandrum sativum L.* seeds, locally sourced and cultured in South West of France. The oil is obtained by mechanical pressing of the fruits using twin-screw extrusion technology, a gentle eco-extraction process without solvent to ensure the protection of the bioactive compounds, protection of the environment, and product safety (10).

**Fig. 1:**
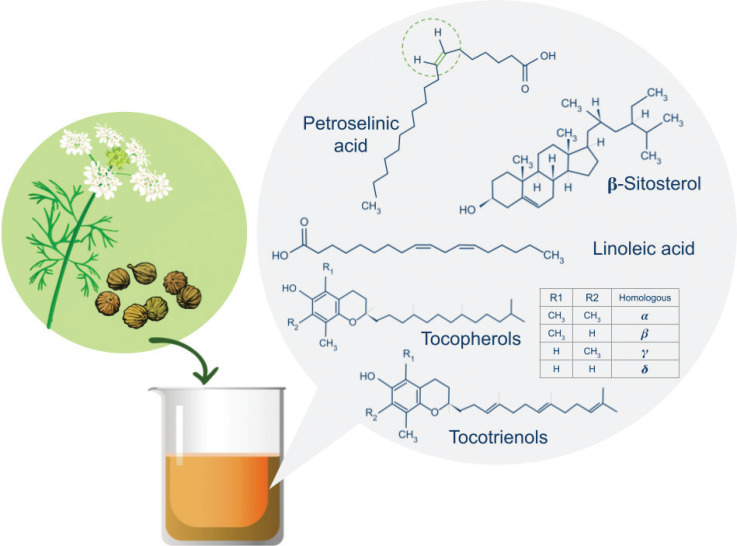
Major components of coriander seed oil.

The fatty acid composition of the virgin coriander oil is characterized to contain 60–75% of petroselinic acid (C18:1n-12) as the major component (Fig. 1). Petroselinic acid is an uncommon monounsaturated omega-12 positional isomer of oleic acid, with a rare 6-position of the double bond mainly found in the seeds from Apiaceae crops. This fatty acid has demonstrated that it can reach tissues and decrease the arachidonic acid concentration, which could provide it with anti-inflammatory properties (11). Virgin coriander oil also contains at least 12% of linoleic acid (Fig. 1) and its derivatives, which play a central role in the structure and function of the *stratum corneum* permeability barrier. Linoleic acid is the most abundant fatty acid in the epidermis. Importantly, it is also the precursor to ceramides, a major component of the extracellular lipid matrix that forms the *stratum corneum* permeability barrier (12).

CSO is also a natural source of phytosterols and tocols (13, Fig. 1). Phytosterols and, particularly, β-sitosterol, the most abundant of them, are known for having anti-inflammatory action (14, 15). Tocols, that is, tocopherols and tocotrienols, are fat-soluble vitamin E isomers that can protect the oil from oxidative reactions. Tocols are also known to have the antioxidant capability, protecting the skin by scavenging free radicals, stabilizing the membranes of cells, reducing the number of apoptotic cells, and minimizing the activation of nuclear factor-kappaB (NF-κB) (16).

Based on the mechanisms supporting sensitive skin and the knowledge on coriander and its oil components, the effects of CSO have already been investigated *in vitro* on inflammation and nociception. CSO regulates the NF-κB activation involved in inflammation and the transient receptor potential ankyrin 1 (TRPA1) activation involved in nociception (17). Therefore, it was consistent to study the soothing effects of CSO *in vivo*. The objective of this pilot clinical study was to evaluate the soothing effects of CSO on women having sensitive skin and whose skin was submitted to mechanical and chemical stress.

## Materials and methods

### Design and protocol

**Fig. 2:**
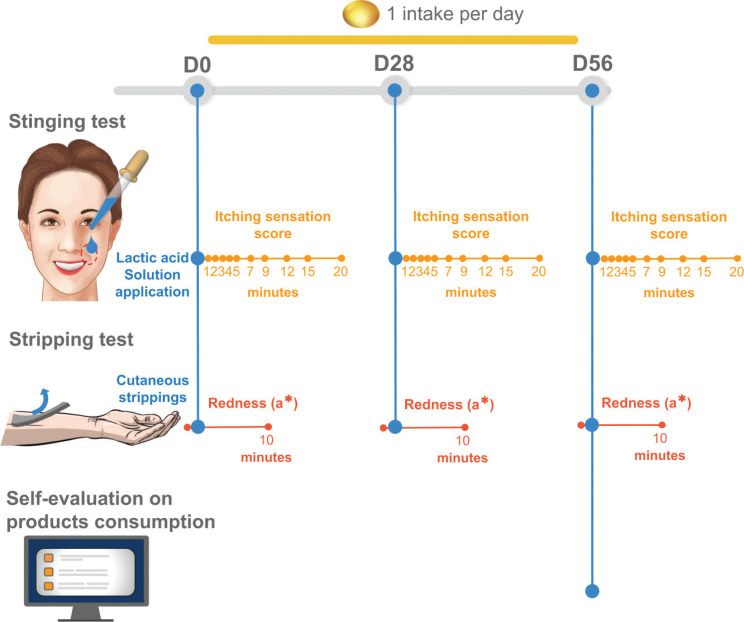
Study design.

The study was designed as a pilot randomized, parallel-group, double-blind trial to test the effect of a daily dose of 200 mg of CSO *versus* placebo for 56 days. Study assessments were done on days 0 (D0, baseline), 28 (D28), and 56 (D56) at the Complife facilities in the Milan area (Italy) (see Fig. 2 for study design). The study was approved by an independent ethics clinical investigation committee in July 2019 (Complife Italia Prot. no: E.HU.027-0090.02.003L _2019/194) and conducted in full accordance with the principles of the 1975 Declaration of Helsinki, revised in 2000. All participants gave written informed consent before participation.

### Intervention

The test products were in the form of soft gelatin (from bovine) opaque capsules containing sunflower oil (400 mg for the placebo group and 200 mg for the CSO group) with the addition of 200 mg of CSO (Sepibliss^TM^) for the active group, for a total weight of 400 mg. Volunteers consumed 1 capsule daily in the morning for 56 days.

### Participants

Female participants, aged between 18 and 65 years, were included in the study with the following criteria: Caucasian ethnicity; phototype I to IV (according to Fitzpatrick classification); with sensitive and reactive skin, that is, showing positive answer from 3-moderate to 4-severe to stinging test with lactic acid at 10% (see details in paragraph “lactic acid-induced stinging test” below); not recently involved in any other similar study; willing to use only the products to be tested throughout the study period; willing not to use similar products that could interfere with the product to be tested; willing not to vary the normal daily routine (i.e., lifestyle and physical activity); under effective contraception if women of childbearing potential and not expected to be changed during the trial; aware of the study procedures and having signed an informed consent form; and willing to avoid intensive exposure to UV rays throughout the study. The exclusion criteria were pregnant/breastfeeding female or planning a pregnancy during the study period, positive history of atopy or hypersensitive skin areas that could interfere with the functionality of the product under study, under systemic pharmacological treatment, under local pharmacological treatment on the skin area monitored during the test, with congenital or acquired immunodeficiency, under treatment with food supplements that could interfere with the functionality of the product under study, having skin alterations on the monitored area that could interfere with the functionality of the product under study, and with known or suspected sensitization to one or more test formulation ingredients.

### Outcome assessment

The outcomes reported below were assessed under controlled environmental conditions (temperature 18–26°C and room humidity = 40–60%). Subjects were left to acclimatize to ambient conditions for 15–20 min before the measurements.

#### Skin stripping

At each experimental time (D0, D28, and D56), skin redness (transient and not harmful skin alterations) was induced through the removal of serial layers of stratum corneum (20 cutaneous strippings with surgical tape) on the volar surface of the forearm of each subject to evaluate the product’s soothing activity in terms of efficacy in preventing the skin redness reaction induced by the skin stripping.

Evaluation of skin redness was carried out just before and 10 min after skin stripping at each experimental time. The measurement of the red component of the skin color (a* value) was carried out using Colorimeter CM-700d (Konica Minolta).

#### Lactic acid-induced stinging test

At each experimental time (D0, D28, and D56), a stinging feeling (transient and not harmful skin discomfort) was induced through the application of a 10% lactic acid solution on the nasolabial fold of each subject to evaluate the product’s calming effect in terms of efficacy in preventing the skin itching sensation induced by the lactic acid solution. Itching/stinging severity was assessed by the volunteers at different time points (e.g., 1, 2, 3, 4, 5, 7, 9, 12, 15, and 20 min) after the application of lactic acid solution using a defined 4-point clinical scale (1: no itching/stinging sensation, 2: mild sensation, 3: moderate sensation, 4: severe sensation).

#### Self-assessment questionnaire

At the end of the study (D56) and before any induction of stress, subjects were asked to express their personal opinion on the products being tested by answering a questionnaire about product acceptability and perceived effects. The items targeted wordings associated with unpleasant sensations described for sensitive skin and soothing feelings. The statements presented to the subjects were as follows: ‘taking the product reduces skin sensitivity’, ‘taking the product intake reduces skin reactivity’, ‘taking the product reduces skin redness’, ‘after using the product, you feel your skin is more protected’, ‘after using the product, you feel your skin is more tolerant’, ‘after using the product, you feel your skin is more relieved’, and ‘after using the product, you feel your skin is more repaired’. Possible answers were ‘completely agree’, ‘agree’, ‘disagree’, or ‘completely disagree’.

### Statistical analysis

As this was a pilot clinical study on CSO, no calculation of the number of subjects was performed. Guidelines for pilot studies recommend recruiting between 10 and 40 subjects per group (18). Moreover, clinical studies evaluating the efficacy of supplementation with plant oils, such as evening primrose or borage oils, for eczema were also mostly performed on 10–40 subjects per group (19, 20). Therefore, 30 subjects per group were included. Analysis was performed on the per protocol (PP) population. Missing data were not replaced.

For the redness parameter, as a* depends upon individual variability of skin color, delta (a*) was calculated using the values obtained before and after stripping with the following formula: delta (a*) = a* (after stripping) − a* (before stripping) at each time D0, D28, and D56. For each group, the mean and standard error of the mean (SEM) were calculated. Statistical analysis was performed with repeated measures analysis of variance (ANOVA), followed by Tukey’s test for pairwise comparisons.

For the stinging parameter, integrative score during the kinetics of measurement, peak intensity and duration of reaction were calculated for each subject with the following formulas:

- Integrative score = Sum [(tm) – (previous tm)] × [score (tm) + score (previous tm)] ÷ 2 for tm = time of measurement 1, 2, 3, 4, 5, 7, 9, 12, 15, 20 min
- Peak intensity = score (time = 4 min) as the maximal score was reached at this time
- Duration of reaction = time during which score was greater than or equal to two

Mean and SEM were then calculated for each group at D0, D28, and D56. Within-groups statistical analysis was performed with ANOVA or ANOVA on ranks according to the normality of data.

Furthermore, as evaluation by stinging score is subjective and depends on the skin reactivity at baseline and perception of each subject, the differences between CSO and placebo groups were evaluated on the changes at D28 and D56 from baseline (D0) for the above three parameters. Adjusted between-group differences and corresponding *P*-value were calculated with the use of repeated-measurements analysis of covariance (ANCOVA), using baseline values (D0) as covariates, and presented as least-square mean (LSmean) and 95% CI, overall and at D28 and D56 specifically.

For the self-assessment questionnaire, the percentage of positive answers (‘completely agree’ or ‘agree’) were calculated for the placebo and CSO groups and compared with chi-squared test.

## Results

### Study participants

Ages and skin phototypes are detailed in Table 1. No difference between groups was observed.

**Table 1:** Descriptive data of the population at baseline (D0).

	Placebo group	CSO group
Age (years)	42.1 ± 2.9	47.4 ± 2.0
Skin phototype	12 II, 11 III, 7 IV	1 I, 10 II, 16 III, 3 IV

Values were expressed as mean ± SEM.

**Fig. 3:**
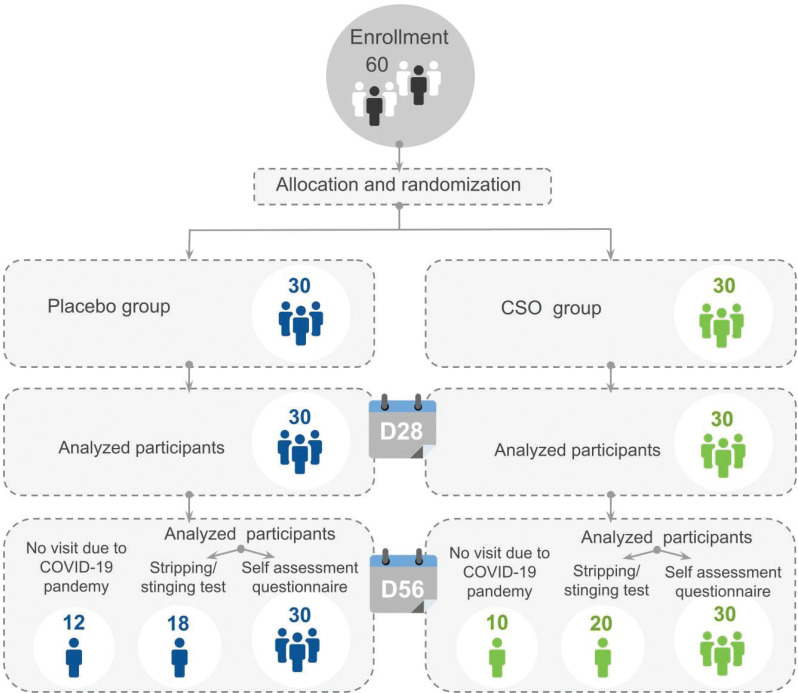
Flow chart of the clinical study.

All data were available on D0 and D28. Due to the COVID-19 pandemic, the trial was not completed by all the subjects on D56: 20/30 subjects completed the study in the CSO group and 18/30 subjects completed it in the placebo group for stress tests. Nevertheless, all subjects could continue with product use until D56 to answer the final self-assessment (see Fig. 3 for study flow chart).

### Redness induced by stripping stress

**Fig. 4:**
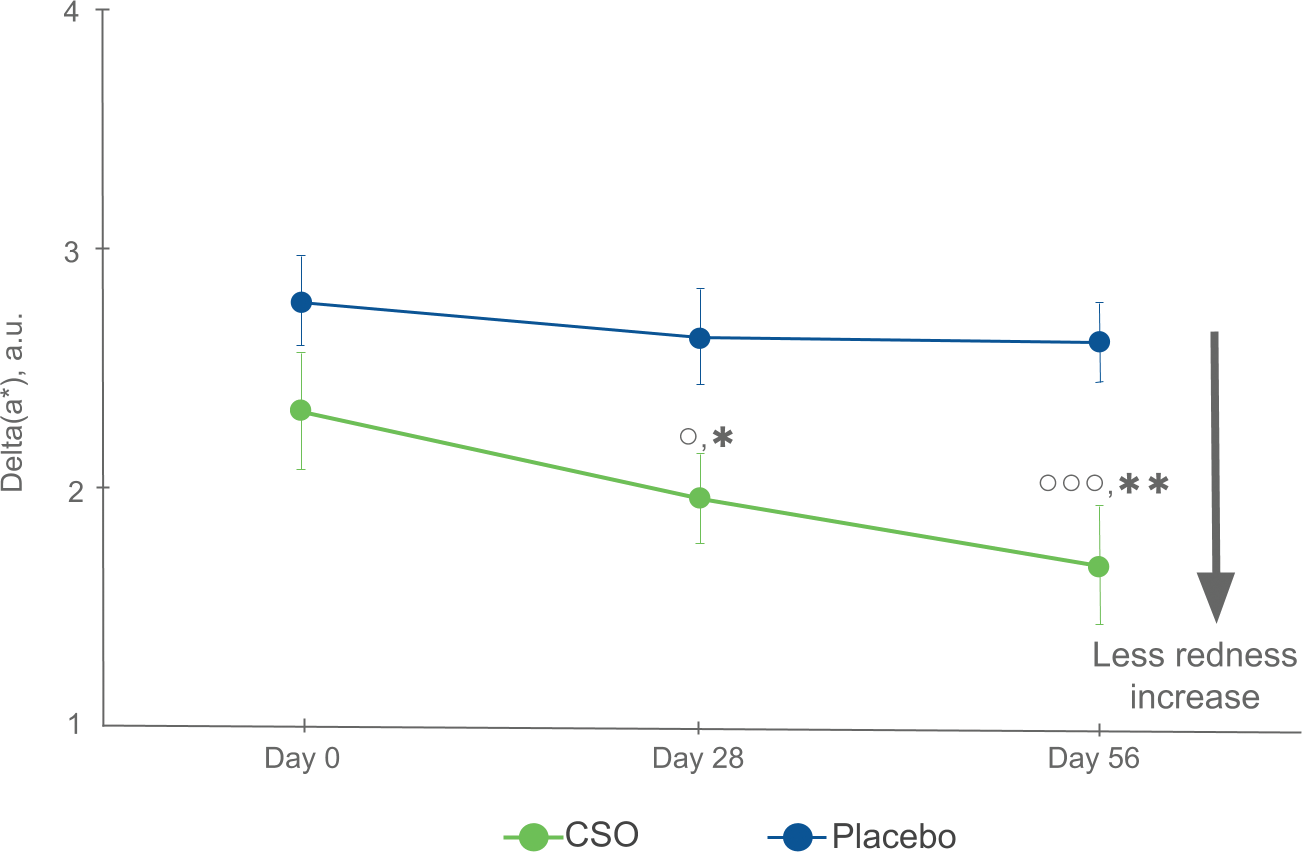
Delta (a*) before and 10 min after stripping, a.u. Values are expressed as mean ± SEM. For the placebo group, the number of subjects (*n*) was *n* = 30 at D0 and D28 and *n* = 18 at D56. For the CSO group, the number of subjects (*n*) was *n* = 30 at D0 and D28 and *n* = 20 at D56. °*P* < 0.05, °°°*P* < 0.001 *versus* D0. *P < 0.05, **P < 0.01 *versus* placebo.

Redness was evaluated before and 10 min after stripping stress on D0, D28, and D56, and delta (a*) before and after stripping was calculated at each time (Fig. 4). No statistically significant differences were observed in the placebo group over time (day 0: 2.78 ± 0.19 a.u., *n* = 30; day 28: 2.63 ± 0.20 a.u., *n* = 30; day 56: 2.61 ± 0.17 a.u., *n* = 18). A statistically significant decrease was observed in the CSO group over time (D0: 2.32 ± 0.25 a.u., *n* = 30; D28: 1.95 ± 0.19 a.u., *P* = 0.044 *versus* D0, *n* = 30; D56: 1.67 ± 0.25 a.u., *p* < 0.001 *versus* D0, *n* = 20). Furthermore, there were significant differences between groups at D28 (*P* = 0.019) and D56 (*P* = 0.005).

### Integrative score of itching/stinging sensation after stinging stress

**Fig. 5:**
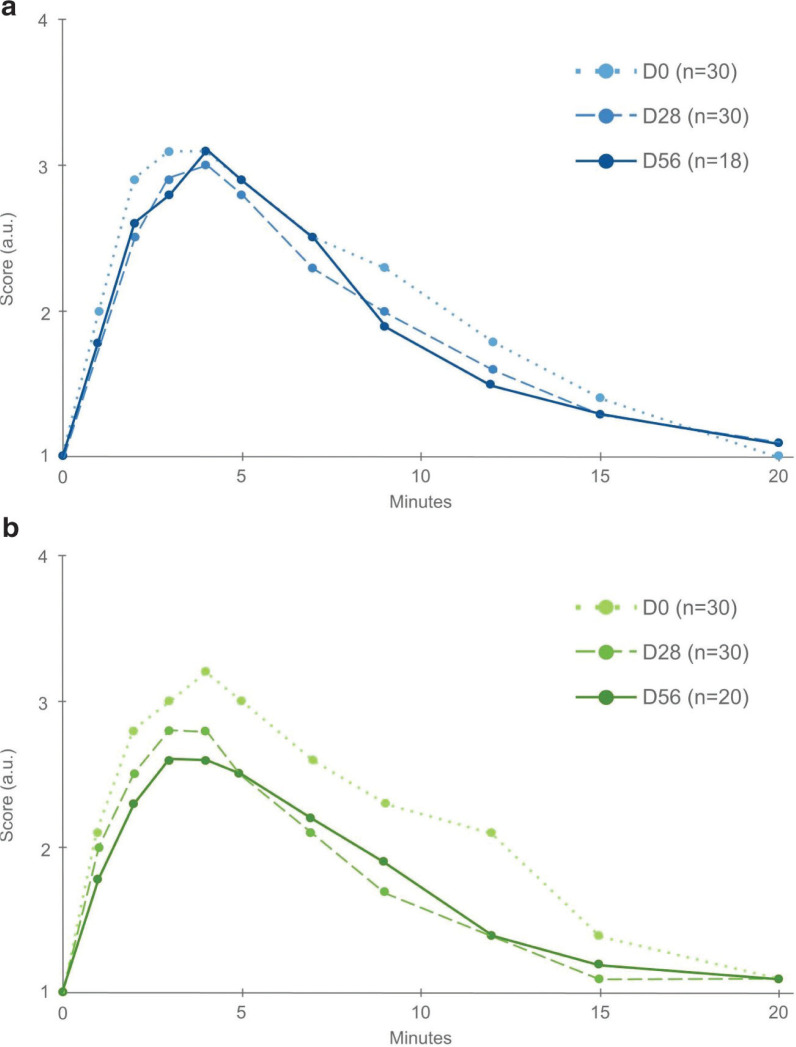
(a) Evolution of itching score in the placebo group, a.u. (b) Evolution of itching score in the CSO group, a.u.

Itching/stinging sensation was evaluated after stinging stress on D0, D28, and D56, and integrative scores on the 20 min evaluation were calculated (Fig. 5a, b and Table 2).

**Table 2:** Integrative score of stinging reaction.

Product, Day of visit	*n*	Integrative score (a.u.)	Differences between groups on changes		
			Overall	At D28	At D56
**Placebo** D0 D28 D56	30 30 18	40.3 ± 1.8 37.0 ± 1.6, ns 37.6 ± 2.5, ns	−4.4 (−7.7, −1.2) *P* = 0.008	−3.5 (−7.4, 0.3) *P* = 0.071	−5.3 (−10.2, −0.5) *P* = 0.032
**CSO** D0 D28 D56	30 30 20	41.8 ± 1.8 34.2 ± 1.3, *P* = 0.001 33.9 ± 2.0, *P* = 0.006			

Descriptive data of the population at D0, D28, and D56: values are expressed as mean ± SEM; *n,* number of subjects; *P versus* D0 for intragroup analysis. Differences between groups on changes: values are expressed as LSmean (95% CI), corresponding *P*-value between groups. ns, not significant.

Within-group analyses showed that no change was observed in the placebo group, whereas a significant decrease was shown in the CSO group (Table 2, *P* = 0.001 at D28 and *P* = 0.006 at D56).

The overall change in the CSO group was significantly greater than that in the placebo group (−4.4, 95% CI = −7.7 to −1.2, *P* = 0.008). A trend was observed at D28 (−3.5, 95% CI = −7.4 to 0.3, *P* = 0.071), which was confirmed by a significant difference at D56 (−5.3, 95% CI = −10.2 to −0.5, *P* = 0.032).

### Peak stinging reaction score after stinging stress

The peak stinging reaction score was reached 4 min after the application of lactic acid solution. Scores at 4 min at D0, D28, and D56 were calculated (Table 3).

**Table 3:** Peak stinging reaction score.

Product, Day of visit	*n*	Peak stinging reaction score (a.u.)	Differences between groups on changes		
			Overall	At D28	At D56
**Placebo** D0 D28 D56	30 30 18	3.1 ± 0.1 3.0 ± 0.1, ns 3.1 ± 0.2, ns	−0.5 (−0.8, −0.1) *P* = 0.007	−0.2 (−0.5, 0.0) *P* = 0.095	−0.7 (−1.3, −0.1) *P* = 0.018
**CSO** D0 D28 D56	30 30 20	3.2 ± 0.1 2.8 ± 0.1, *P* = 0.009 2.6 ± 0.2, *P* = 0.017			

Descriptive data of the population at D0, D28, and D56: values are expressed as mean ± SEM; *n,* number of subjects; *P versus* D0 for intragroup analysis. Differences between groups on changes: values are expressed as LSmean (95% CI), corresponding *P*-value between groups. ns, not significant.

Within-group analyses showed that no change was observed in the placebo group, whereas a significant decrease was shown in the CSO group (Table 3, *P* = 0.009 at D28 and *P* = 0.017 at D56).

The overall change in the CSO group was significantly greater than that in the placebo group (−0.5, 95% CI = −0.8 to −0.1, *P* = 0.007). A trend was observed at D28 (−0.2, 95% CI = −0.5 to 0.0, *P* = 0.095), which was confirmed by a significant difference at D56 (−0.7, 95% CI = −1.3 to −0.1, *P* = 0.018).

### Duration of itching/stinging reaction after stinging stress

Reaction duration during the stinging test was also evaluated for each subject by calculating the time during which the score stayed above or equal to two (Table 4). Within-group analyses showed that no change was observed in the placebo group, whereas a significant decrease was shown in the CSO group (Table 4, *P* = 0.0008 at D28 and *P* = 0.005 at D56).

**Table 4:** Stinging reaction duration.

Product, Day of visit	*n*	Stinging reaction duration (min)	Differences between groups on changes		
			Overall	At D28	At D56
**Placebo** D0 D28 D56	30 30 18	10.9 ± 0.8 10.3 ± 0.8, ns 9.7 ± 1.2, ns	−2.0 [−4.0, −0.1] *P* = 0.038	−1.8 [−3.9, 0.3] *P* = 0.089	−2.3 [−4.8, 0.3] *P* = 0.083
**CSO** D0 D28 D56	30 30 20	12.4 ± 0.7 9.3 ± 0.8, *P* = 0.0008 8.9 ± 1.1, *P* = 0.005			

Descriptive data of the population at D0, D28, and D56: values are expressed as mean ± SEM; *n,* number of subjects; *P versus* D0 for intragroup analysis. Differences between groups on changes: values are expressed as LSmean (95% CI), corresponding *P*-value between groups. ns, not significant.

The overall change in the CSO group was significantly greater than that in the placebo group (−2.0, 95% CI = −4.0 to −0.1, *P* = 0.038). Trends were observed at both D28 (−1.8, 95% CI = −3.9 to 0.3, *P* = 0.089) and D56 (−2.3, 95% CI = −4.8 to 0.3, *P* = 0.083).

### Self-assessment questionnaire

**Fig. 6:**
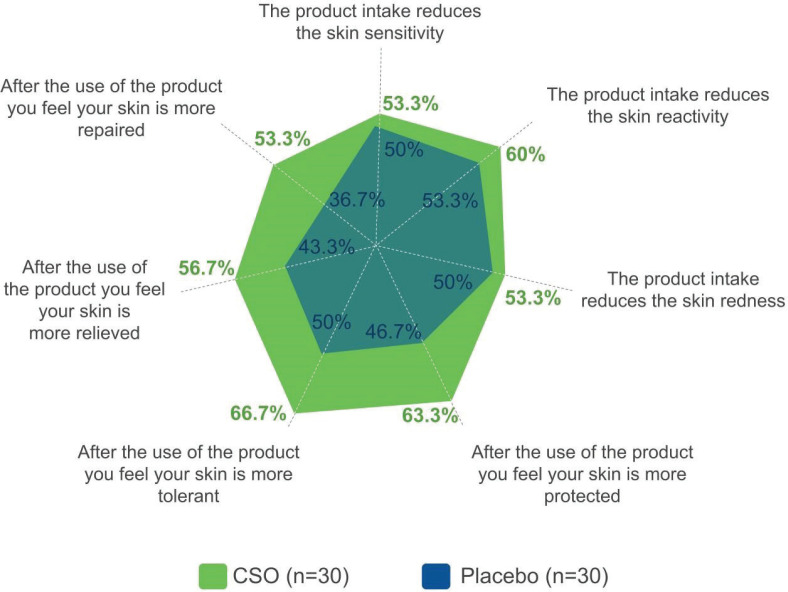
Self-assessment questionnaire (D56).

The questionnaire (Fig. 6) was completed by all the subjects (*n* = 30 for both groups). All the answers were in favor of the CSO group, even without reaching the significance (chi-squared test). Most notably, the subjects in the CSO group found their skin more protected, tolerant, relieved, and repaired than that in the placebo group. As an example, for the statement ‘after using the product, you feel your skin is more relieved’, 56.7% of the subjects in the CSO group answered ‘completely agree’ or ‘agree’, compared with 43.3% in the placebo group.

## Discussion

The objective of this pilot clinical trial was to evaluate the soothing effect of CSO on women with sensitive skin when reactivity was induced by mechanical (stripping) or chemical stress (lactic acid). Supplementation with CSO regulates the increase of redness induced by successive strippings on the volar surface of the forearm of volunteers. CSO also regulates itching sensations induced by lactic acid application on the nasolabial fold of subjects, measured either by integrative score on the measurement session, by peak intensity or by reaction duration. This effect was observed with a daily dose of 200 mg from 28 days of consumption. From a user perspective, the efficacy of CSO was perceived by the subjects as better than the placebo, especially on the criteria of repair, relief, tolerance, and protection. These results reinforce the previous *in vitro* data obtained on the regulation of inflammation and nociception (17), and they highlight CSO as a promising soothing active ingredient for sensitive skin. To the best of our knowledge, this is the first time that CSO has demonstrated a soothing effect *in vivo*. In the first intention, as it was a pilot study, only women with sensitive skin were recruited to ensure as much as possible a homogeneous group for inclusion. Furthermore, the prevalence of sensitive skin is higher in women than that in men. These results need to be confirmed in other human trials and also in men.

Thanks to their specific fatty acid profiles, plant oils are recognized as good candidates to influence skin barrier function and play a role in improving skin conditions. Supplementation with other plant oils has already been tested in clinical studies, not on sensitive skin but preferably on atopic eczema. The most tested ones were borage and evening primrose oils because they are a source of gamma-linolenic acid, an omega-6 fatty acid known to decrease inflammation. The last meta-analysis on clinical studies looking at the effect of these oils on atopic eczema identified 19 studies on evening primrose oil and eight studies on borage oil, however, without concluding on a positive effect of these oils (20). Other plant oils have been tested to a lesser extent. For example, hemp seed oil, rich in alpha-linolenic acid, improved skin dryness and itching sensations in atopic dermatitis (21). Supplementation of flaxseed oil showed evidence of reduced skin sensitivity generated by nicotinate irritation in Western women (22). Topical application of plant seed oils was also tested and showed promising and different effects according to their composition and skin conditions (23).

Like some other plant oils, CSO is characterized by a specific fatty acid profile, and more particularly thanks to petroselinic acid, which accounts for 60–75% of the oil. It has already been demonstrated that it can reach tissues and decrease arachidonic acid concentration (11), and thus, petroselinic acid could contribute to the anti-inflammatory and soothing effect of CSO. Petroselinic acid is an uncommon monounsaturated omega-12, which is mainly found in the seeds from Apiaceae crops. It could be of interest to test the supplementation by other plant oils rich in petroselinic acid or petroselinic acid itself on skin functions to better understand the role of this fatty acid in the relief of sensitive skin.

CSO also contains linoleic acid, which contributes to the structure and barrier function of the epidermis. It could also partly explain the beneficial effect observed with CSO. Nevertheless, sunflower oil, which was used as a placebo in this pilot study, also mostly contains linoleic acid and did not show any beneficial effect in the study. This is also true for safflower oil tested on skin sensitivity in parallel to flaxseed oil (22). This oil, which contains more than 70% linoleic acid, induced a slight improvement of skin roughness but no effect on skin sensitivity (22). The contribution of linoleic acid is then far from being proven.

CSO is also a natural source of phytosterols and tocols, which are recognized to have anti-inflammatory and antioxidant properties, respectively. The oil contains about 6.70 g/kg of phytosterols and 500 mg/kg of tocols (13). Even if it is higher than most of the vegetable oils in phytosterols (1–5 g/kg), this should not explain the observed beneficial effect of CSO *versus* sunflower oil, which also contains these compounds. It could, therefore, be of interest to analyze further which components contribute to the observed effects and by which mechanisms.

Moreover, investigations of additional parameters such as quantification of inflammatory mediators and/or lipid composition of *stratum corneum* in future clinical studies may be worth pursuing to go further in the analysis of the soothing mechanisms of CSO.

The soothing effects observed with CSO in women with sensitive skin are very promising. Further studies are now needed to confirm them and to understand the associated mechanisms of action.
